# Efficacy, Safety, and Long-Term Follow-Up Results of EUS-Guided Transmural Drainage for Pancreatic Pseudocyst

**DOI:** 10.1155/2013/924291

**Published:** 2013-03-10

**Authors:** Shin Kato, Akio Katanuma, Hiroyuki Maguchi, Kuniyuki Takahashi, Manabu Osanai, Kei Yane, Toshifumi Kim, Maki Kaneko, Ryo Takaki, Kazuyuki Matsumoto, Tomoaki Matsumori, Katsushige Gon, Akiko Tomonari

**Affiliations:** Center for Gastroenterology, Teine-Keijinkai Hospital, Sapporo 006-8555, Japan

## Abstract

*Background and Aim*. EUS-guided transmural drainage (EUS-GTD) is now considered a minimally invasive and effective alternative to surgery for drainage of symptomatic pancreatic pseudocysts. However, the technique is rather difficult, and sometimes serious complications occur to patients undergoing this procedure. We retrospectively evaluated efficacy, safety, and long-term follow-up results of EUS-GTD for pancreatic pseudocyst. *Methods*. Sixty-seven patients with pancreatic pseudocyst who underwent EUS-GTD from April 2000 to March 2011 were enrolled. We retrospectively evaluated (1) technical success, (2) clinical success, (3) adverse event of procedure, and (4) long-term follow-up results. *Results*. Total technical success rate was 88%. Ninety-one percent of external drainage, 79% of internal drainage, and 66% of puncture and aspiration only achieved clinical success. There was only one case with an adverse event, perforation (1.5%). The case required emergency operation. Total recurrence rate was 23.9%. Median follow-up period was 33.9 months. The recurrence rates in the cases of stent remaining, spontaneously dislodged, removed on schedule, external tube removal, and aspiration only were 10.0%, 12.5%, 42.9%, 50%, and 0%, respectively. *Conclusion*. EUS-GTD is a relatively safe and effective therapeutic method. However, further analysis should be done by larger series to determine the method of EUS-GTD for pancreatic pseudocyst.

## 1. Introduction

 Endoscopic ultrasonography guided transmural drainage (EUS-GTD) is now a widely reported and established procedure for drainage of symptomatic pancreatic pseudocyst. The technical success rate of this procedure has been reported to be more than 90% on the other hand, complication rate is less than 5% [1–5]. Severe adverse events such as bleeding or perforation sometimes occur, but in general, EUS-GTD has been recognized as a relatively safe and effective therapeutic method and is performed as a common procedure in many institutes. However, some factors including the long-term outcome, appropriate period of stenting, and timing of removing the stent have not been determined yet.

## 2. Patients and Methods

 Patient characteristics are described in Table 1. Sixty-seven patients with pancreatic pseudocyst who underwent EUS-GTD from April 2000 to March 2011 were enrolled. Indications of EUS-GTD at our center are as follows: symptomatic pancreatic cysts, infection or hemorrhage, rapid increase in size, and no communication with MPD. In this study, 3 cases after acute pancreatitis (type 1) had walled off necrosis. Abdominal computed tomography and transabdominal ultrasonography had been taken in all cases before EUS-GTD. 

All procedures were performed using curved linear array EUS (GF UCT-240, GF UCT-260 Olympus Medical Systems, Tokyo, Japan). The used needles are EchoTip Ultra 19G (Cook Medical Inc., Winston Salem, NC, USA), GW, Visiglide 0.025 inch (Olympus Medical Systems), Jagwire 0.035 inch (Boston Scientific Japan, Tokyo, Japan), dilation catheter, Needlecut 3V (Olympus Medical Systems), and Soehendra biliary dilation catheter (Cook Medical Inc.). We used Pigtail nasobiliary catheter (6,7 Fr, Olympus Medical Systems. 7.5 Fr, Boston Scientific Japan) for external drainage and 7 Fr double pigtail catheter (Olympus Medical Systems) for internal drainage.

 We evaluated (1) technical success rate of initial drainage, (2) Clinical Success rate, (3) adverse event of procedure, and (4) long-term follow-up results after EUS-GTD.

## 3. Technique of EUS-GTD 

All procedures were performed in the fluoroscopy unit under given intravenous sedative agent. Following steps were used to perform EUS-GTD.

**Figure 1:**
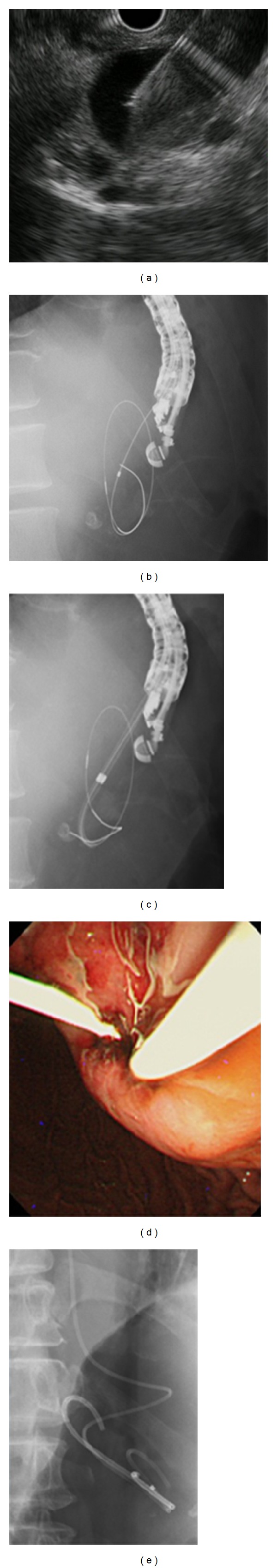
(a) Pancreatic pseudocyst was punctured with EchoTip 19 G needle. (b) Fluoroscopy image showed that the guide wire was passed through the needle into the lumen of cyst. (c) After electrocautery, Soehendra dilation catheter was inserted into the lumen for dilatation. (d) and (e) 7 Fr double pigtail stent and 6 Fr ENBD pigtail catheter were placed.

The cystic lesion of pancreas was examined with ultrasonographically, and doppler assessment of gastric or duodenal wall was performed to confirm the absence blood vessels at the puncture site. Then, pancreatic pseudocyst was punctured with EchoTip 19G needle (Figure 1(a)) and the GW was passed through the needle into the lumen of the cyst. The position of GW was confirmed by ultrasonography and fluoroscopy (Figure 1(b)). After placing the GW, electrocautery was applied with NeedleCut3V, and dilation was achieved with a Soehendra biliary dilation catheter (Figure 1(c)). After dilatation, 2nd GW was inserted through Soehendra biliary dilation catheter, and plastic stent and drainage tube were placed under the guidance of GW.  7Fr double pigtail stent was used for internal drainage and 6Fr ENBD pigtail catheter for external drainage (Figures 1(d) and 1(e)).

 We mainly chose external drainage. However, in cases where patients were likely to remove the tube by themselves or there was no infected fluid in the cyst, we chose internal drainage. In some cases with pseudocyst with small size and no infected fluid, we performed only puncture and aspiration. From April 2010, we inserted both internal and external drainage tube at one procedure session.

## 4. Results

### 4.1. Technical Success

Total technical success rate was 88.1% (59/67) (Table 2). Technical success rates of transgastric approach and transduodenal approach were 88.3% and 75.0%, respectively. Depending on the procedure, the success rates of external drainage, internal drainage, both internal external drainage, and punctured and aspiration only were 83.7%, 95%, 100%, and 100%, respectively. Reasons for technical failure were difficulty in advancing the GW or its displacement in 2 cases, difficulty in achieving dilation in 4 cases, and difficulty with drainage tube placement in 2 cases. In these failed cases, we performed puncture and aspiration only. 

### 4.2. Clinical Success

In our series, clinical success was defined as cases in which initial EUS-GTD contributed to cyst shrinking over 10 mm or improvement of clinical symptoms.  Table 3 shows that clinical success rate of external drainage was 91.7% (33/36), internal drainage was 78.9%, both internal and external drainage was 66.7% (2/3), and puncture and aspiration only was also 66.7% (6/9). Total clinical success rate was 83.4% (56/67). In the failed 3 cases of external drainage, 1 case had additional multiple stenting which contributed to cyst shrinking, and 2 cases had operation. The failed 4 cases of internal drainage had added external tube drainage and all cases achieved cyst shrinking consequently. The failed 1 case of external and internal drainage had additional multiple stenting which was effective. In the failed 3 cases of puncture and aspiration only, each case had either operation, ESWL, or EPS (Table 4). 92.5% of the cases (62/67) achieved improvement by EUS-GTD.

### 4.3. Adverse Event of Procedure

**Figure 2:**
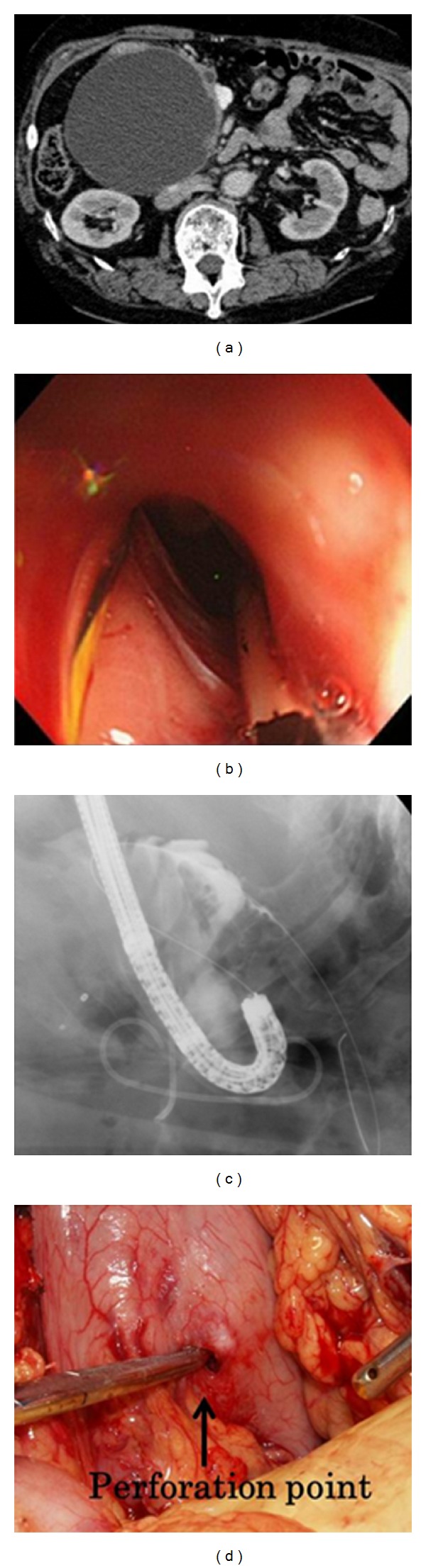
(a) Type III pancreatic pseudocyst. (b) The edge of omentum was observed through the hole dilated by balloon. (c) Leakage of contrast medium and advancing of the guide wire into the abdominal cavity were observed. (d) Perforation point was clearly confirmed by operation.

The rate of adverse event was 1.5% (1/67). There was one case with perforation in our series. The perforation case had a type III pancreatic cyst (Figure 2(a)). Perforation occurred during balloon dilatation for multiple stents placement. After balloon dilatation, we recognized leakage of contrast medium and advancing of the GW into the abdominal cavity (Figures 2(b) and 2(c)). This case required surgical operation (Figure 2(d)).

### 4.4. Long-Term Follow-Up Results (Table 5)

In the 46 cases which finally achieved clinical improvement by EUS-GTD and were observed for more than 1 year, overall recurrence rate was 23.9% (11/46). Median follow-up period of the above cases was 33.9 months. Final stent conditions were as follows: 40 cases had internal drainage (ID), 4 cases had external tube removal (stent free), and 2 cases had only puncture and aspiration. Stent was still remaining in 10 cases of ID and 1 of them had recurrences (10%). In 16 cases of ID, stent was dislodged spontaneously, and 2 of them had recurrences (12.5%). In 14 cases of ID, stents were removed on schedule and 6 of them had recurrence (42.9%). 2 cases of aspiration only and external drainage tube removal had recurrences (20%). 

 We also analyzed the factors of recurrence by comparing recurrence group (*n* = 11) and nonrecurrence group (*n* = 35) (Table 6, Fischers test, *P* value <.05 was considered statistically significant); however, no factors were significant to determining recurrences. 

## 5. Discussion

 The technical success rate of EUS-GTD has been reported to be more than 90% in several studies [1–5]. In our series, the technical success rate was 88.1%. The technical success rate of external drainage was slightly low (83.7%), but the rates of internal drainage and both internal and external drainage were relatively high (95% and 100%, resp.). Clinical success rate was 83.4%, and by adding extra EUS-GTD procedure, 92.5% of the cases achieved clinical improvement. Our result also supported that EUS-GTD is an effective procedure for pancreatic pseudocyst. 

 It is stated in previous studies that some cases with walled off necrosis after acute pancreatitis need not only tube drainage but also debridement. The success rate of EUS drainage for pancreatic abscess or necrosis was lower compared with clear fluid pancreatic pseudocyst [6]. One of the recent studies also reported that multiple transluminal gateway technique for EUS guided drainage was clearly effective for walled off necrosis of pancreas [7]. In our series, 3 cases had walled off necrosis; however, all improved by only conservative external drainage without debridement. 

 Severe adverse events such as perforation and bleeding sometimes occur in EUS-GTD. The rate of adverse event was reported to be less than 5% [5]. Our series also had 1 case with perforation and that case needed operation. In our case, perforation occurred when balloon dilatation was performed. Balloon dilatation method has been thought to be a safer method compared to using electrocautery [8]. However, if the cyst wall of pancreas and gastric wall are not adhered to each other, the risk of perforation is clearly increased. Pancreatic cyst of our perforation case was type III, and this seemed to be the main cause of perforation. In our institute, first step drainage for type III cyst is endoscopic transpapillary drainage except in cases with severe clinical symptoms such as cyst infection or abdominal pain. In 14 cases out of 26 type III cysts, we performed transpapillary drainage (ENPD) first, but the procedure was not effective. In 3 cases, we failed to place ENPD and performed transmural drainage only. There were 9 cases in which transmural drainage was directly performed due to severe cyst infection or remarkable abdominal pain. EUS-GTD was performed directly in the case with perforation due to cyst infection. 

Since the rate of adverse events in our series is low (1.5%), it can be said that EUS-GTD is a relatively safe procedure for pancreatic pseudocyst drainage. Although EUS-GTD is a safe and effective therapy method for drainage of pancreatic pseudocyst, prospective studies which compare EUS-GTD to surgery to evaluate the long-term outcome are required [9]. 

 We had chosen external drainage mainly and in some cases, we chose internal drainage. Recently, we insert both internal and external drainage tube in one session of procedure. Several current studies recommended insertion of both internal and external drainage tube by using double guidewire technique [10, 11]. There is no prospective study comparing efficacy between both internal and external tube placement at once or external tube or internal tube only. In our series, it was necessary to replace external drainage tube to internal drainage tube in 26 cases and to replace internal drainage tube to external drainage tube in 1 case. Since recannulating the pseudocyst is sometimes cumbersome, the combination placement of internal and external drainage tube using double guidewire technique will be an effective and reasonable method for pancreatic pseudocyst drainage. 

 Total recurrence rate in our study was 23.9%. The current another study also reported that recurrence rate of fluid collection was 13.4% [12]. The reasons for recurrence may include not only stent condition but also any background of pancreas condition. We also analyzed several factors for recurrences; however, no particular factors were significant in our series. The result was not shown to be significant. The reason for this may be that this was a single center study, and was performed retrospectively. A larger prospective series study is required for detecting the factors for recurrence. 

## 6. Conclusion

EUS-GTD is a relatively safe and effective therapeutic method. Therefore, EUS-GTD can be the first choice for therapy of pancreatic pseudocyst. However, further analysis should be done by a larger series to check the efficacy and safety and to determine the method of EUS-GTD for pancreatic pseudocyst.

## Figures and Tables

**Table 1:** Clinical characteristics of the patients.

Gender M : F	50 : 17
Median age, yr (range)	56 (31**~**85)
Median cyst size, diameter in mm (range)	72 (19–186)
Cyst location	
Head	23
Body and tail	44
Classification	
Type I*: acute pancreatitis	15
Type II: acute exacerbation of chronic pancreatitis	14
Type III: retention cyst	26
Type IV: pancreatic fistula after pancreatic surgery	12

*3 cases in type I had walled off necrosis.

**Table 2:** Technical success rate of initial drainage.

Access route	Stomach	88.3% (53/59)
	Duodenum	75% (6/8)
Success rate of procedure	External drainage	83.7% (36/43)
	Internal drainage	95% (19/20)
	Internal and external drainage	100% (3/3)
	Puncture and aspiration only	100% (1/1)

Total*		88.1% (59/67)

*The rate was defined as the cases that the procedures accomplished as planned successfully. Failed 8 cases had puncture and aspiration later.

**Table 3:** Clinical success rate.

Initial drainage	Rate
External drainage	91.7% (33/36)
Internal drainage	78.9% (15/19)
Internal and external drainage	66.7% (2/3)
Puncture and aspiration only	66.7% (6/9)*

Total	83.4% (56/67)

*8 cases (internal or external drainage failed) were included.

**Table 4:** Management of failed cases in initial drainage.

Initial drainage	Failed cases	Additional therapy	
External drainage (*n* = 36)	3	Multiple stents	1
		Repuncture*→operation	1
		Operation	1
Internal drainage (*n* = 19)	4	Add external drainage	4
External and internal drainage (*n* = 3)	1	Multiple stents	1
Puncture and aspiration only (*n* = 9)	3	ESWL	1
		EPS	1
		Operation**	1

ESWL: extracorporeal shock wave lithotripsy. EPS: endoscopic pancreatic stent. *CT guided—cyst drainage. **Operation for an adverse event caused by EUS-CD procedure.

**Table 5:** Recurrence rate and stent condition.

Recurrence rate 23.9% (11/46) *n* = 46			
Final drainage	Stent condition*		Recurrence
*Internal drainage* (*n* = 40)	Still remaining	10	1 (10%)
Single stent (*n* = 34)	Spontaneous dislodged	16	2 (12.5%)
Multiple stent (*n* = 6)	Scheduled removal	14	6 (42.9%)
*Puncture and aspiration only* (*n* = 2)	Stent-free	2	0 (0%)
*External tube removal* (*n* = 4)	Stent-free	4	2 (50%)

*The duration of internal drainage: 0.5~97.1 (median 20) months.

**Table 6:** Evaluation of factors for recurrence.

	Recurrence (11)	Non-recurrence (35)	*P* value
Patients characteristics			
Male	9	29	0.7064
Age < 50	4	15	0.9756
Age ≥ 70	4	8	0.6197
Location of cyst, Ph	3	11	0.9089
Diameter of cyst ≥ 10 cm	2	6	0.7064
Infection	5	14	0.7485
Procedure			
Internal tube drainage	9	31	0.9466
External tube removal	2	2	0.4685
Puncture, aspiration only	0	2	0.9706
Stent remaining	1	9	0.4551
Initial clinical failure	2	1	0.2732
Multiple stenting	1	5	0.9744
